# Interactions of Overweight, Poor Oral Health, and Stress Related to Chronic Disease in an Aging Population

**DOI:** 10.1155/2010/614814

**Published:** 2010-12-08

**Authors:** Julia W. Albright

**Affiliations:** Division of Health Programs, International Relief & Development, Inc., 1621 North Kent Street, Arlington, VA 22209, USA

## Abstract

The prevalence of excess body mass (XBM), poor oral health (POH), and stress in a secluded population of aged (≥60 years) Hmong immigrants was surveyed. The findings were related to the prevalence of diabetes in the same population. Diabetes was associated separately with POH (OR 2.4; CL 1.3, 4.2) or with XBM (OR 2.5; CL 1.4, 4.8). The association of diabetes with the combination of XBM and POH was striking (OR 5.1; CL 3.4, 7.5); that apparent synergism has not been fully appreciated. We describe a mechanism that explains the synergism. The concept of “thrifty genotype” is a plausible explanation of XBM in the elderly Hmong immigrants and possibly the current older Laotian population. POH is common among elderly Laotians as it is in most developing countries. We conclude that synergism of XBM and POH significantly elevates the prevalence of diabetes among aging populations and probably other age groups as well.

## 1. Introduction

Among several factors that relate to the deterioration of health associated with aging, one that is high on the scale of health problems is excess body weight (XBM) and accompanying chronic inflammatory diseases (CIDs). The prevalence of XBM/obesity and CIDs begins to increase significantly in late mid-life and rises steadily until near the end of the life span (sixth and seventh decades in the human, approximately). Thereafter, the prevalence declines gradually, reflecting, in part, mortality of those most susceptible to disease. 

Two accepted causes of obesity are (a) supercharged diets high in calories and processed fats and low in fiber and (b) inadequate physical exercise. Diet control and regular physical activity are advocated to prevent and lessen XBM. Familiar ideas about weight reduction through exercise need to be reevaluated in light of recent work which indicates that vigorous exercise alone cannot effect loss of excess weight [1]. There are other recognized causes of XBM and CIDs in addition to poor diet and inadequate exercise, such as poor oral health (POH) [2], stress [3], and environmental pollutants [4].

The purpose of this paper is to describe some recent findings from a study of the interactions between XBM, stress, and POH. The study involved a relatively secluded population of elderly in which a supercharged diet was not a dominant factor. We found that the frequency of diabetes, as a sentinel CID, is strongly associated with the simultaneous occurrence (interaction) of XBM and POH. To our knowledge, that apparent synergism has not been fully recognized.

## 2. Material and Methods

### 2.1. Study Population

The study involved an elderly population (≥60 years of age) of Hmong people who immigrated to the Central Valley of California subsequent to the conflict in Vietnam. The Hmong people are indigenous to the highlands of Laos and Southern China. Those immigrants are now an aged population. They have remained relatively secluded and have retained their language and cultural attributes including their dietary preferences (green leafy vegetables, fish, and chicken) Their access to professional medical and dental care has been somewhat limited.

### 2.2. Study Survey

A survey of 877 subjects (477 women, 400 men), aged 60 years and over, was conducted. Nearly all of the elderly, original immigrants in the area were included in the survey. Each older subject was interviewed by a volunteer project associate (PA). Their weight and height were measured and used to calculate the body mass index (BMI). Each subject was asked to complete a questionnaire designed for this survey and translated into the language of the subjects. The PAs were recruited by a collaborating organization, The Merced Lao Family Community, Inc. (H. Vang, Director). They were Laotian-Americans who speak the language and dialect of the elders and are familiar with the culture. Nearly all of the subjects were unable to speak or understand English. The PAs were selected and trained/instructed by the author to conduct the interview and administer the questionnaire. The study protocol and questionnaire were reviewed by a human subjects review committee (Ethical review Committee, Inc., Independence, MO) and approved (April 7, 2009). The purpose and plan of the study were explained to each subject by a PA, and informed consent was obtained. All of the elderly subjects contacted by the PAs agreed to participate in the survey. That high degree of participation was a reflection of (a) the esteem of The Merced Lao Family Community among the elderly Hmong, and (b) acceptance of the PAs all of whom speak the language and understand the culture of the elderly Hmong people.

The questionnaire was composed of 35 items (questions). It was designed by the author who used the short form health survey (SF-36) [5] and recommended modifications [6] as a guide. Questions were designed to reveal (a) current age, age at immigration, year of immigration, and years of residence in CA; (b) frequency and types of exercise; (c) self-assessed status of health and list of personal health problems including disabilities; (d) evidence of chronic stress; (e) access to physicians, and dentists; (f) preferred foods, routine daily diets and knowledge of nutrition. 

Data were entered in coded format into a secure computer. Subsequent statistical analyses were performed with the aid of a statistical package. Care was taken to insure the privacy of information concerning each subject.

## 3. Results

### 3.1. Age Distribution

Table 1 shows the distribution of the subjects by age. The majority immigrated when they were between ages 30–50 years. A few were 60 years or older. The majority have resided in California for more than 10 years, some for more than 30 years.

### 3.2. BMI/Risk Categories and Years of Residence in California

We have employed the scheme recommended by the World Health Organization for Asian populations [7] to categorize the BMI data of the elderly Hmong subjects. That system rates a BMI of 18.5–22.9 kg/m^2^ as being of “acceptable risk”, 23–27.4 kg/m^2^ as being the “elevated risk” (overweight) range, and >27.5 as being the obese, high risk, category.

Table 2 records the distribution of the BMI data, both in the acceptable risk and the elevated risk/obese categories, by years of residence in California. The data were treated by Chi squared trend analysis for evidence that overweight/obesity increased with duration of residence in California. The analysis revealed no evidence of a population shift toward XBM (*P *>  .1). The same trend analysis was applied separately to the data from the subjects either in the 60–69-year group or the 70–79-year group (Table 1). The outcome was the same, and there was no shift toward XBM with duration of residence in California (*P *>  .1 in both cases). Those results are consistent with the conclusion, based on responses to the questionnaire, that the Hmong immigrants have shunned obesogenic, local foods.

### 3.3. Prevalence of POH, Stress, and Diabetes among the Elderly Hmong Immigrants

Data gathered by interview and questionnaire revealed that disorders of body mass and oral health, along with stress and diabetes, were frequent in the elderly Hmong subjects. The prevalence of the disorders was found to be as follows: diabetes: 24%, POH: 41%, stress: 27%, and XBM: 87%. Those distressing numbers are well above the frequencies of the disorders in the general, elderly US. population except, possibly, for stress which has been estimated to affect nearly half of the adult population [8].

### 3.4. Association of XBM, POH, and Stress with Diabetes

Diabetes was treated as a sentinel CID and used to estimate the probabilities that XBM, POH and stress might be related causally to CIDs (Table 3). The relationship between XBM and diabetes was considered first. The likely role of XMB was suggested by an odds ratio (OR) of 2.5 (see Table 3). Considered alone, POH was related to the presence of diabetes (OR 2.4). Stress alone appeared unrelated to diabetes: there were no subjects who were both stressed and diabetic in the absence of XBM or POH.

Inspection of the data revealed that 60% of subjects with diabetes also evinced XBM and POH. A Chi squared test of the odds of diabetes being associated with XBM and POH concurrently, compared to the sum of the odds of diabetes occurring with XBM or POH separately, was conducted (Table 4); the observed data were used to calculate the OR (Table 3). The large OR (5.1) provides compelling evidence that the presence of diabetes is more likely in subjects suffering both XBM and POH than in subjects with only one of those afflictions.

### 3.5. Overweight and Obesity in Lao PDR and Other Developing Countries

The high frequency of XBM in the elderly Hmong subjects coupled with the evidence that a significant change in their diet has not occurred subsequent to their immigration, raised questions about body weight among the native Laotian people. 

Data obtained from the WHO internet site [9] pertaining to Lao PDR and several other developing countries are presented in Table 5. The BMI data for all subjects in our survey were reorganized according to the conventional scale (overweight: >25.0; obesity: >30 kg/m^2^). The data from all seven countries included both urban and rural populations. Only data for the female gender were available for comparison. The age groups were matched as closely as the WHO data would allow. 

Although the comparisons allowed by the discrepant matching of age groups are imperfect, two conclusions can be drawn from Table 5. First, a relatively high prevalence of XBM, including almost 15% obesity, exists in the population of Lao PDR. The close agreement of the figures from the Hmong immigrants of Central Valley and the population of Lao PDR is fortuitous. However, the BMI of the Hmong immigrants resembles that of the Lao population. The second conclusion is that the relatively high prevalence of XBM among the Laotian people is not unique although it appears to be significantly higher than in neighboring countries (Cambodia and Vietnam). Both Congo and Malawi report relatively high prevalence of XBM, possibly higher than Laos and significantly higher than other African countries. The African countries shown in Table 4 were picked at random; there are probably others with relatively high prevalence of XBM.

## 4. Discussion

### 4.1. Body Mass

XBM creates extra work for the heart, lungs, and kidneys compared to an acceptable (normal) body mass. XBM is also a significant risk factor for CIDs (also called metabolic diseases). White fat masses that accumulate in the visceral region become infiltrated by proinflammatory macrophages and, together with engorged adipocytes, release cytokines and other inflammatory substances.

Subsequent events may lead to disorders such as hyperglycemia, insulin resistance, fat deposition in blood vessels, hypertension, and other life-threatening aberrations.

### 4.2. Stress

The associations between stress, XBM and stress, and CIDs have been well documented [10–12]. Moderate stress that heightens alertness and prepares the body for action is a necessary aspect of self-defense and survival. Too much stress, chronic or prolonged, is potentially pathogenic. Chronic stress such as that experienced in conflict, combat, or grievous socioeconomic conditions, frequently leads to disease (obesity, diabetes, cardio- or cerebro-vascular crises, respiratory disorders such as sleep apnea, and others). Note also an intriguing report [13] that indicates obesity can spread in a social network wherein weight gain in one individual is accompanied by weight gain in others. The stress found in the elderly Hmong immigrants can be attributed to (a) military experience as many of the subjects were once soldiers or soldiers' families, (b) cultural maladaptation reflecting years of residence in an unfamiliar environment, and (c) socioeconomic stress as most of them have lived with insufficient income. However, we found no association of stress with the occurrence of diabetes in the older Hmong subjects.

### 4.3. Oral Health

A link between POH and CIDs has been reported. For example, a large survey of the US. population [2] revealed that subjects over 45 years of age who suffered from severe periodontitis were 2.3 times more likely to have metabolic syndrome than persons in good oral health. There is also an evident association of POH and type 2 diabetes [14] and insulin resistance [15].

### 4.4. Synergisms: Diabetes in Relation to Combination of XBM and POH

The results of the survey of the elderly Hmong revealed the expected relationship between XBM and diabetes and POH and diabetes. However, analysis of the data for the presence of diabetes in subjects in whom XBM and POH were comorbid produced an odds ratio and probability that compel the conclusion of synergism. That is, diabetes is present much more frequently in subjects who are overweight and suffer both XBM and POH than in subjects who either are overweight or have POH but are not afflicted by both. The bidirectional relationship between periodontal disease and diabetes mellitus has been reviewed recently [16]. The evidence indicates that periodontitis occurs more frequently and is more severe in patients who have diabetes than in those who do not. Other evidence indicates that poor glycemic control is found frequently in patients with periodontitis. The association between diabetes and POH among the aged Hmong in this study could have resulted either from the diabetes leading to or exacerbating POH or vice versa. Presumably, the appearance of XBM, POH, and diabetes in subjects having all three, disorders could have occurred in any order of the three; one sequence beginning with POH and endotoxemia. 

A mechanism underlying the XBM/POH synergism can be envisioned. Several gram-negative bacteria appear in the oral cavity in association with edentulism, caries, gingivitis, and/or periodontitis. Endotoxins released by those bacteria enter the bloodstream via lesions in the mouth and by translocation across the “leaky” mucosal epithelium associated with diets deficient in protein and fiber. Toll-like receptors (TLRs) are well represented on cells in the oral cavity and intestinal epithelium [17, 18]. TLR4, a component of the complex receptor for endotoxins, is present in abundance on both adipocytes and macrophages located in visceral fat [19, 20]. In addition to endotoxins, several oral pathogens (*Porphyromonas gingivalis, Bacteroides forsythus*) release protein adhesins (components of fimbriae) that are recognized by TLR4 and TLR2 [20, 21]. Both endotoxins and protein adhesins stimulate macrophages to secrete proinflammatory cytokines (IL-1, IL-6, and TNF*α*). Endotoxins and adhesins stimulate adipocytes to secrete inflammatory molecules. The most exaggerated effect of endotoxin stimulation occurs when cocultures of adipocytes and macrophages are stimulated with endotoxin [22]. Strong expression of genes associated with inflammation is evident both in adipocytes and macrophages. The exaggerated expression of proinflammatory genes results from endotoxin activation of TLR4 receptor complex and subsequent endoplasmic reticulum (ER) stress in a variety of types of cells, including adipocytes and macrophages. ER stress and the unfolded protein response lead to efficient translocation to the nucleus of the potent transcription factors, NF*κ*B and AP1 [23]. The effects of IL-1, IL-6, and TNF*α* on cells in various organs lead to inflammation and CIDs.

### 4.5. Overweight and Chronic Inflammatory Diseases in Developing Countries

The WHO has sounded the alarm concerning the scope of the world's oral health deficiencies and needs [24]. So far, oral health has not appeared near the top of the global public health “popchart”. One of the difficulties in dealing with the problem in developing countries is the paucity of dentists. A look at World Health Statistics, 2009 [25], reveals that in the majority of countries there is less than 1 dentist per 20,000 population. In many cases there are fewer than 10 dentists in the entire country.

The high prevalence of XBM in Lao PDR (Table 5) is probably a relatively recent occurrence. Data published by the WHO [9] indicates that a rapid increase in the BMI of women (and men) occurred between the years 2000 (overweight 12%, obesity 2% among women) and 2005 (overweight 55%, obesity 15%). It may be concluded that at the time of emigration of most of the elderly Hmong enrolled in this survey, XBM in Lao was almost nonexistent. It should be stressed that (a) the WHO data pertain to the whole population of Lao of which the Hmong are approximately 7% and (b) the Hmong are indigenous to the mountainous regions of Laos to which access can be difficult. Therefore, the Hmong are likely to be underrepresented in health surveys conducted in Laos. Although we lack accurate data, it seems safe to conclude that the Hmong who immigrated some years prior to their participation in this survey were not overweight.

Given the sustained preference for the foods they consumed in Laos, we suspect that the gain in body mass that occurred after the Hmong immigrated was not the consequence of devouring Western foods or sudden indolence. Rather, we are inclined to invoke the “thrifty gene” concept [26, 27] to explain the gain in mass. 

That acquisition must have occurred within a few years after arriving in California as there is evidence against it having been a continuing process (Table 2). The thrifty gene hypothesis proposes that the metabolic machinery works toward creating fat during frequent or extended periods of food scarcity. Once adapted, the metabolism does not readily adjust when there is an abundance of nutrients. Thus, it continues to produce stores of fat resulting in increased body mass. In time, presumably, a new equilibrium is established and increase in body mass does not continue indefinitely. In the case of the Hmong immigrants (and other similar populations) the actions of a thrifty genotype (phenotype) together with widespread oral pathology could readily account for the high prevalence of diabetes and other chronic diseases.

We recognize that this survey suffers from the limitations of every cross-sectional study and that it is hazardous if not foolish to attempt to determine “causes” from such a survey. Ultimately, it will be necessary to conduct a longitudinal study if it can be done. In the meantime, we believe there are other ways to decide whether or not the conclusions posed here have merit.

## 5. Conclusion

It is common knowledge that the older age group (60 plus years) is the most rapidly increasing segment of the world's population. That segment of the population is also most prone to be overweight/obese and to evince the highest frequency of poor oral health. The latter comprises missing teeth, edentulism, periodontitis, poorly fitting dentures, and other maladies. POH can worsen or even cause XBM. It would seem that attention to oral hygiene and correction of existing dental/oral problems should be national health priorities in developing countries. Attention to population oral health could be the cheapest and most effective, preventive approach to reducing the dangers of overwhelming numbers of chronically ill elderly.

## Figures and Tables

**Table 1:** Categorization of elderly Hmong subjects by chronological age.

Age in years (by decade)	Number of subjects	Percent of total subjects
60–69	533	61
70–79	257	29
80–89	65	7
≥90	22	3

	877	100

**Table 2:** BMI/Risk category of subjects by years of residence in California.

BMI/Risk Category	Number of subjects by years of residence^1^				
	<4	10–14	15–19	20–24	25–29
18.5–22.9	22	26	39	37	44
≥23.0	76	129	186	164	142

^1^Category of 5–9 years and 30 + years omitted, too few subjects.

**Table 3:** Odds ratios of associations between diabetes excess body mass (XBM), poor oral health (POH), and the combination of the latter two (XBM/POH).

Association: diabetes with		Confidence limits		
	Odds ratio	Lower	Upper	*P*
XBM	2.5	1.4	4.8	<.01
POH	2.4	1.3	4.2	<.01
XBM/POH	5.1	3.4	7.5	<.001

**Table 4:** The association of diabetes with concurrent XBM and POH compared to its association with the sum of XBM and POH occurring separately^a^.

Diabetes	XBM & POH		
	Concurrent	Separate	Totals
Yes			
Observed	123	78	201
Expected	78	123	

No			
Observed	70	225	295
Expected	115	180	

Totals	193	303	496

*Null hypothesis:* no association of diabetes with concurrent presence of XBM & POH			
*Null hypothesis rejected, P < .001*			

^a^The data are presented in the format of a contingency table that was used for Chi squared analysis. The data for OR calculation are in the “observed” rows.

**Table 5:** Prevalence of excess body mass among Lao immigrants (California) and in several countries of Asia and Africa.

			Prevalence (%)^1^	
Country (year of report)	Gender	Age group	BMI range (kg/m^2^)	
			≥25^2^	≥30^2^
Hmong of Central Valley, CA (2009)	Female	60+	58.8	14.6
Lao PDR (2005)	Female	30+	54.7	14.5
Cambodia (2006)	Female	40–49	17.0	2.4
Vietnam (2005)	Female	30+	13.0	0.5
Congo (2005)	Female	40–49	43.5	17.0
Malawi (2005)	Female	45–49	19.3	4.8
Namibia (2007)	Female	40–49	47.4	25.7
Uganda (2005)	Female	30+	24.2	2.0

^1^Urban and rural data combined.

^2^BMI ≥ 25: elevated risk of chronic diseases; ≥30: high risk of chronic diseases (acceptable risk: 18.5–24.9 kg/m^2^).
